# Assessing the Clinical Effectiveness of an Exergame-Based Exercise Training Program Using Ring Fit Adventure to Prevent and Postpone Frailty and Sarcopenia Among Older Adults in Rural Long-Term Care Facilities: Randomized Controlled Trial

**DOI:** 10.2196/59468

**Published:** 2024-07-18

**Authors:** Sheng-Hui Tuan, Lin-Hui Chang, Shu-Fen Sun, Chien-Hui Li, Guan-Bo Chen, Yi-Ju Tsai

**Affiliations:** 1 Institute of Allied Health Sciences College of Medicine National Cheng Kung University Tainan Taiwan; 2 Department of Rehabilitation Medicine Cishan Hospital Ministry of Health and Welfare Kaohsiung Taiwan; 3 Department of Physical Medicine and Rehabilitation College of Medicine Kaohsiung Medical University Kaohsiung Taiwan; 4 Department of Occupational Therapy College of Medicine National Cheng Kung University Tainan Taiwan; 5 Department of Physical Medicine and Rehabilitation Kaohsiung Veterans General Hospital Kaohsiung Taiwan; 6 School of Medicine College of Medicine National Yang Ming Chiao Tung University Taipei Taiwan; 7 Department of Internal Medicine Kaohsiung Armed Forces General Hospital National Defense Medical Center Kaohsiung Taiwan; 8 Department of Physical Therapy College of Medicine National Cheng Kung University Tainan Taiwan

**Keywords:** exergame, Ring Fit Adventure, sarcopenia, frailty, long-term care, multicomponent training

## Abstract

**Background:**

Frailty and sarcopenia are geriatric syndromes of increasing concern and are associated with adverse health outcomes. They are more prevalent among long-term care facility (LTCF) users than among community dwellers. Exercise, especially multicomponent and progressive resistance training, is essential for managing these conditions. However, LTCFs, particularly in rural areas, face challenges in implementing structured exercise programs due to health care professional shortages. Moreover, older adults often become bored with repetitive exercise training and may lose interest over time. The Nintendo Switch Ring Fit Adventure (RFA) exergame is a novel exergame that combines resistance, aerobic, and balance exercises and offers a potential solution by boosting motivation in an immersive manner and reducing staff intervention needs.

**Objective:**

We aimed to evaluate the clinical effectiveness of an exergame-based exercise training program delivered via RFA (exergame-RFA) in improving muscle mass and functional performance among older adult LTCF users.

**Methods:**

This was a randomized controlled trial conducted from August 2022 to September 2023 and involved older adult LTCF users (aged ≥60 y) in rural southern Taiwan. Participants were randomized into an intervention group (exergame-RFA plus standard care) or a control group (standard care alone). The intervention, conducted seated with *arm fit skills* and trunk control exercises using the RFA, lasted 30 minutes twice weekly over 12 weeks. The primary outcomes measured were the Study of Osteoporotic Fractures index (serving as an indicator of frailty status) and the diagnostic criteria for sarcopenia (appendicular skeletal muscle mass index, handgrip strength, and gait speed). The secondary outcomes included functional performance (box and block test as well as maximum voluntary isometric contraction of the dominant upper extremity), muscle condition (muscle thickness measured using ultrasonography), activities of daily living (Kihon checklist), health-related quality of life (Short Form Health Survey-36), and cognitive function (brain health test). We used an intention-to-treat analysis, incorporating a simple imputation technique in statistical analysis. A mixed ANOVA, with time as a within-participant factor and intervention as a between-participant factor, was used to compare the training effects on outcomes.

**Results:**

We recruited 96 individuals, of whom 60 (62%) underwent randomization. Of these 60 participants, 55 (92%) completed the study. Significant group×time interactions were observed in the intervention group in all primary outcomes (all *P*<.001, except *P*=.01 for handgrip strength) and most secondary outcomes, including maximum voluntary isometric contraction of the biceps (*P*=.004) and triceps brachii (*P*<.001) muscles, biceps muscle thickness measured using ultrasonography (*P*<.001), box and block test (*P*<.001), Kihon checklist (physical function: *P*=.01, mood status: *P*=.003, and total: *P*=.003), and brain health test (*P*<.001).

**Conclusions:**

The exergame-RFA intervention significantly improved muscle mass, strength, and functional performance among older adult users of rural LTCFs, offering a novel approach to addressing frailty and sarcopenia.

**Trial Registration:**

ClinicalTrials.gov NCT05360667; https://clinicaltrials.gov/study/NCT05360667

**International Registered Report Identifier (IRRID):**

RR2-10.3389/fmed.2022.1071409

## Introduction

### Background

Global aging is intensifying due to rapid socioeconomic advances and longer lifespans. It is predicted that, by 2025, Taiwan will evolve into a super-aged society, with >20% of its population being aged ≥65 years [1]. This increase in the older adult population brings multiple health challenges, including the effects of aging, chronic diseases, cognitive decline, malnutrition, diminished physical fitness, and psychosocial issues. Aging and inactivity lead to decreases in muscle mass, structure, and strength [2], with individuals aged >50 years losing muscle mass at a rate of 1% to 2% annually [3]. This contributes to sarcopenia, a gradual decline in muscle mass, strength, and physical performance that occurs as individuals age [4], as well as frailty, a state of heightened vulnerability due to the decline in the reserve and function of multiple physiological systems [4].

Frailty and sarcopenia are significant concerns globally, profoundly influencing older adults’ physical capabilities, health risks, quality of life, and longevity [5,6]. In Taiwan, frailty and sarcopenia affect 17.6% [7] and from 4.1% to 9.3% [8] of community-dwelling older adults, respectively. Notably, these conditions are more prevalent among individuals who have been institutionalized at long-term care facilities (LTCFs). Frailty rates can reach 79.4% in nursing homes [9]. Sarcopenia rates vary from 17.7% to 73.3% in long-term nursing facilities [10] and from 22% to 87.1% in day care centers [11]. The higher prevalence of these conditions among older adults in LTCFs is linked to their advanced age, more severe health problems, and increased dependence on assistance for activities of daily living (ADLs) [11].

The current gold standard for managing frailty and sarcopenia focuses on preserving skeletal muscle mass and maintaining muscle strength [12]. Resistance training (RT), particularly progressive RT (PRT), combined with multicomponent exercises, is supported by moderate- to high-quality evidence [13,14]. This evidence highlights their effectiveness in increasing muscle mass, strength, and physical performance in older adults with frailty and sarcopenia [14,15]. When it comes to exercise training, it should be consistently performed at a slightly challenging level of intensity. However, resource limitations, particularly in staffing, present considerable challenges to systematically implementing regular exercise programs in LTCFs [16], especially those in rural areas. Exergames, which are video games necessitating whole-body player engagement, could address the aforementioned issues. They provide real-time interactivity [17] as well as engaging, multisensory environments, facilitating immersive experiences through extensive body movements [18]. The gamification and enticing environments of exergames have been shown to motivate older adults, enhancing their engagement with physical exercises [19]. As a result, the incorporation of exergames reduces the necessity for extensive staff involvement in interventions, encourages patients to engage in more vigorous activities, and boosts their motivation levels.

### Objectives

While the clinical effectiveness of using exergames among community-dwelling older adults is recognized [20,21], their efficacy within LTCFs remains underexplored. Most research focuses on improvements in health-related quality of life (HRQoL), cognitive functions, and overall functional status [22,23]. The literature mainly discusses exergames designed for aerobic and balance training, with limited incorporation of PRT principles. The introduction of Nintendo’s Ring Fit Adventure (RFA) for the Nintendo Switch marks a significant evolution in exergaming, combining aerobic, strength, and balance training tailored to users’ individual needs [24]. Despite its apparent potential, detailed empirical studies to fully ascertain its benefits are still forthcoming. Our team proposed and initiated the Using an Exergame to Prevent and Postpone the Loss of Muscle Mass, Muscle Strength, and Functional Performance in Rural Elders (EXPPLORE) trial [25], aiming to investigate the feasibility and clinical application of RFA for older adults in rural LTCFs. This study presents the outcomes of this protocol.

## Methods

### Study Design and Participants

This study was a prospective randomized controlled trial conducted from August 2022 to September 2023. The trial was designed in compliance with the SPIRIT (Standard Protocol Items: Recommendations for Interventional Trials) 2013 statement for randomized controlled trials and the CONSORT (Consolidated Standards of Reporting Trials) guidelines and has been registered at ClinicalTrials.gov (NCT05360667) (Multimedia Appendix 1; [26]).

Participants were recruited from LTCFs, including day care centers and nursing homes, in rural areas of Kaohsiung City, Taiwan. The eligibility criteria included (1) being aged ≥60 years, (2) residing in LTCFs or attending LTCFs for at least 1 month, (3) having the ability to understand and communicate in Chinese or Taiwanese, (4) possessing adequate cognitive abilities as determined by the researchers to provide informed consent, and (5) actively participating in the exergame-based exercise and data collection process. In addition, participants needed to be capable of remaining seated for >50 minutes during the training session and able to complete the gait speed measurement. We excluded individuals (1) diagnosed with significant cardiopulmonary diseases; (2) on regular oxygen supplementation; (3) with unmanaged hypertension; and (4) with recent infections, fractures, or other conditions that would contraindicate exercise participation according to the American College of Sports Medicine (ACSM) guidelines [27].

Participants were randomly assigned to the intervention and control groups in a 1:1 ratio. The intervention group received standard care combined with RFA for 12 weeks, whereas the control group continued with the usual care typically provided in LTCFs. In the intervention group, traditional sedentary activities common in LTCFs, such as singing, tabletop games, and gardening, were replaced with RFA training sessions. The duration allocated for activities was kept consistent across both groups.

**Figure 1:**
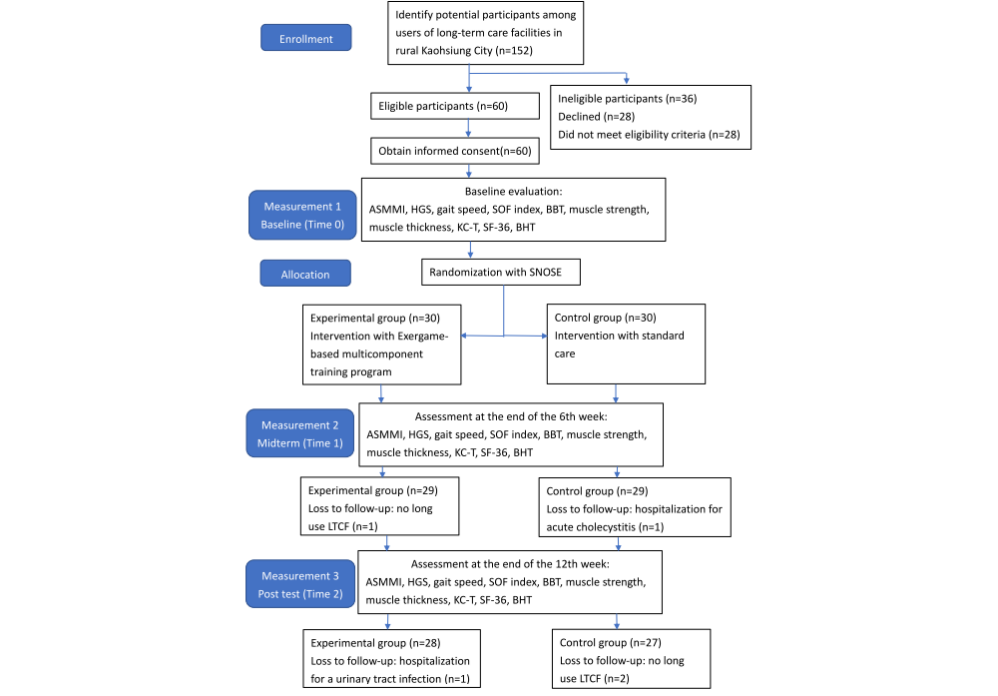
Flowchart of the participant inclusion and data collection processes. ASMMI: appendicular skeletal muscle mass index; BBT: box and block test; BHT: brain health test; HGS: handgrip strength; KC-T: Kihon checklist, Taiwanese version; LTCF: long-term care facility; SF-36: Short Form Health Survey-36; SNOSE: sequentially numbered, opaque, sealed envelope; SOF: Study of Osteoporotic Fractures.

A flowchart detailing the participant inclusion and data collection processes is presented in Figure 1. This study was conducted under the published protocol of the EXPPLORE trial [25].

### Randomization and Blinding

Randomization was achieved using sequentially numbered, opaque, sealed envelopes that contained the group assignment numbers. These envelopes were prepared by an independent individual, and the group assignment numbers were generated by another individual who was not involved in the clinical aspects of the study to ensure blinding to the research proceedings. After verifying eligibility based on the inclusion and exclusion criteria, participants were randomized equally between the intervention and control groups. Given the nature of the study and the distinct characteristics of the interventions, blinding health professionals in the LTCFs and participants to the treatment conditions was not possible. However, to maintain an objective evaluation and minimize bias, the 2 assessors conducting the outcome measurements and the data analyst interpreting the results were blinded throughout the duration of the study.

### Intervention Group Training Program

**Figure 2:**
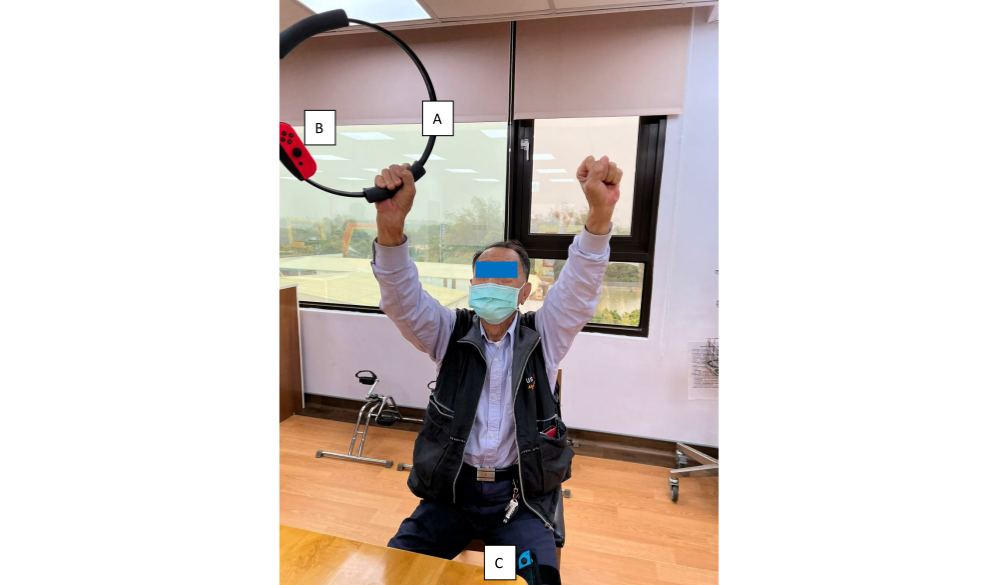
Components of the Nintendo Switch Ring Fit Adventure exergame. (A) Ring-Con, a Pilates ring held by the user; and Joy-Con wireless controllers: (B) right Joy-Con (attached to the Ring-Con) and (C) left Joy-Con (affixed to the leg strap accessory wrapped around the thigh of the player).

The training program (exergame-based exercise training program delivered via RFA [exergame-RFA]) focused on PRT and functional movements, primarily targeting the upper extremities. The RFA system requires a Nintendo Switch console, a Ring-Con (a Pilates ring held by the user), Joy-Con wireless controllers (right Joy-Con, attached to the Ring-Con; and left Joy-Con, affixed to the leg strap accessory wrapped around the thigh of the player), and a display screen (Figure 2).

In RFA’s adventure mode, players move through the game by performing physical activities such as jogging or squatting. Given the increased fall risk among LTCF residents [28], we used RFA’s knee assist mode, making the game more accessible. Enabling knee assist mode provides support when users perform exercises requiring stepping, kneeling, or squatting, making these activities more manageable for players with physical limitations. By adjusting exercise intensity and providing support to the knees, knee assist mode enhances accessibility and inclusivity, allowing a wider range of players to enjoy the game’s fitness benefits. In RFA’s battle mode, players engage in aerobic activities, RT, and yoga exercises involving the full body to advance in the game. Players are rewarded with in-game currency, character customization, and level progression based on exercise volume, encouraging skill improvement.

The exergame-RFA intervention was scheduled twice weekly, with sessions spaced 48 hours apart. Each session lasted 50 minutes: 10 minutes each for warm-up and cool-down exercises and 30 minutes for the main exercise. This was maintained over a 12-week period, with each session overseen by a qualified therapist. Six *fit skills*, which derived from the game’s built-in movement data set and focused on upper extremity and trunk training, were included, with participants using 3 skills during battle mode to defeat opponents. The exergame-based exercises, with targeted muscle groups and joint movements highlighted, are presented in Table S1 in Multimedia Appendix 2 [29-40]. The target intensity of the training was set at a level of 13 (*somewhat hard*) on the Borg rating of perceived exertion (RPE) scale, with progression automatically adjusted by RFA based on each player’s performance (details on RFA content, gameplay steps, and auto-adjustment mechanisms are presented in Table S2 in Multimedia Appendix 2, which outlines the exergame-RFA exercise prescription using the frequency, intensity, time, type, volume, and progression principle formulated by the ACSM [27]).

### Control Group Standard Care Program

For the control group, standard care was provided in accordance with the routine practices of LTCFs. This included group-based activities such as calisthenics (modified for seated positions), horticultural therapy, and sedentary group activities (eg, tabletop games). These sessions, led by a therapist, occurred twice a week, with each session lasting from 30 to 60 minutes, depending on the activity.

### Outcomes Measured

All participants underwent 3 distinct assessments. The initial assessment was conducted at baseline (T0) before randomization. Subsequent evaluations took place at the end of week 6 (midstudy; T1) and at the end of week 12 (after the intervention; T2). The selected primary outcomes for this study were based on the diagnostic criteria for sarcopenia as proposed by the Asian Working Group for Sarcopenia. These included the appendicular skeletal muscle mass index (ASMMI), dominant handgrip strength (HGS), and customary gait speed. In addition, the Study of Osteoporotic Fractures index, serving as an indicator of frailty status, was also designated as a primary outcome.

The secondary outcomes for this study encompassed several dimensions, including muscle strength (indicated by the maximum voluntary isometric contraction [MVIC] of the biceps and triceps brachii muscles, measured using the microFET3 [Hoggan Scientific, LLC]) of the dominant side, muscle morphology (indicated by the thickness of the biceps brachii, quadriceps, and gastrocnemius muscles, measured using a portable LOGIQ e ultrasound device [GE HealthCare] operated by the same operator with gentle pressure in standard positions), manual dexterity of the dominant hand (measured using the box and block test), ADLs (measured using the Kihon checklist), HRQoL [measured using the Short Form Health Survey-36 [SF-36]), and cognitive function (measured using the brain health test). Details of the RFA training process and each outcome measured are available in the published protocol of the EXPPLORE trial [25], and Table S3 in Multimedia Appendix 2 summarizes the instruments and measures implemented for data collection in this study.

### Statistical Analysis

#### Quantitative Data

Statistical analyses were conducted using SPSS for Windows (version 21.0; IBM Corp). Continuous variables were represented as means and SDs, while categorical data were denoted in numbers and percentages. Preliminary checks for data normality and homoscedasticity were performed before executing each analysis. For comparing demographic data between the experimental and control cohorts, appropriate statistical tests, including the chi-square test, 2-tailed *t* test, and Mann-Whitney *U* test, were used, depending on the data distribution characteristics.

To address potential attrition, an intention-to-treat analysis, incorporating a simple imputation technique, was used. Specifically, for any missing data, the last-observation-carried-forward method was used [41]. To assess the effects of the intervention on outcomes over time, a mixed ANOVA was used, designating time as a within-participant variable and the nature of the intervention as a between-participant variable. For any significant interactions or main effects, post hoc assessments were carried out using the Bonferroni test.

#### Sample Size Estimation

Given the absence of a high-quality study evaluating the effects of a comparable intervention involving using RFA on muscle strength, physical activity, and functionality at the time our manuscript was drafted, we based our primary outcome measure (ASMMI) on the findings from a single study that used an augmented reality–based exercise [42]. The effect size observed in the study for the augmented reality–based intervention was 0.71, which is notably substantial [42]. The calculations using G*Power for Windows (version 3.1.9.2; Heinrich Heine University), indicated a requirement for a minimum of 18 participants in each group to detect a difference of 2 SDs in the ASMMI score between the groups, assuming a power of 80% and an α level of 5%. The effect size, situated within the *F* test family, was set at 0.4 [43]. Anticipating a potential dropout rate of 20%, especially considering that older adult LTCF users often have multiple comorbidities, it was deemed prudent to increase the number of participants in each group to 22. Consequently, the recruitment target for the study was set at a minimum of 44 participants.

### Ethical Considerations

This study, conducted in accordance with the Declaration of Helsinki, received approval from the institutional review board of National Cheng Kung University Hospital (B-ER-111-058). Written informed consent was obtained from all participants. The study data were anonymized and deidentified. No compensation was provided to participants.

## Results

### Baseline Characteristics and Adherence to the Study Protocol

From August 2022 to September 2023, a total of 96 older adults from nursing homes and day care centers in rural Kaohsiung City, Taiwan, were recruited, of whom 28 (29%) declined to participate, and 8 (8%) did not meet the eligibility criteria. The remaining 60 participants consented and were randomized: 30 (50%) to the intervention group and 30 (50%) to the control group. Of these 60 participants, 55 (92%) completed the study. Of the 30 participants in the intervention group, 2 (7%) dropped out (n=1, 50% before T1 because they no longer used the LTCFs; and n=1, 50% before T2 because they were hospitalized for a urinary tract infection). Of the 30 participants in the control group, 3 (10%) withdrew (n=1, 33% before T1 because they were hospitalized for acute cholecystitis; and n=2, 67% before T2 because they no longer used the LTCFs). The dropout rates were not significantly different between the groups (*P*=.64).

Table 1 shows the baseline characteristics of the participants. Of the 30 participants in the intervention group, in terms of the frailty and sarcopenia status distribution, 4 (13%) were categorized as *prefrail*, 13 (43%) as *frail*, 16 (53%) as *possible sarcopenia*, 2 (7%) as *confirmed sarcopenia*, and 3 (10%) as *severe sarcopenia*. Of the 30 participants in the control group, 8 (27%) were categorized as *prefrail*, 7 (23%) as *frail*, 24 (80%) as *possible sarcopenia*, 1 (3%) as *confirmed sarcopenia*, and 2 (7%) as *severe sarcopenia*. The average long-term care case-mix system levels were 4.20 (SD 0.79) for the intervention group and 4.40 (SD 0.82) for the control group. A case-mix system categorizes individuals based on their requirements for assistance with ADLs. These levels play a crucial role in determining the suitable level of care and support needed by individuals in LTCFs, ranging from minimal assistance to intensive nursing care [44]. This result indicated that both groups required moderate daily activity assistance. There were no significant baseline differences between the groups, except for the fact that the participants in the intervention group had a lower average HGS.

**Table 1:** Demographic characteristics of the participants at baseline.

Characteristics		Intervention group (n=30)	Control group (n=30)	*P* value^a^	
**Sex, n (%)**					.79
	Male	10 (33)	11 (37)		
	Female	20 (67)	19 (63)		
Age (y), mean (SD)		78.83 (7.71)	78.73 (6.82)	.96	
Height (cm), mean (SD)		152.89 (7.99)	153.88 (8.84)	.67	
Weight (kg), mean (SD)		57.00 (10.05)	61.44 (11.07)	.12	
BMI (kg/m^2^), mean (SD)		24.33 (3.57)	25.86 (3.47)	.12	
Body fat (%), mean (SD)		29.16 (7.82)	32.86 (6.92)	.08	
**BMI group, n (%)**					.88
	Underweight	1 (3)	1 (3)		
	Normal	13 (43)	11 (37)		
	Overweight	13 (43)	16 (53)		
	Obese	3 (10)	2 (7)		
Case-mix system levels, mean (SD)		4.20 (0.79)	4.40 (0.82)	.53	
**Participants below cutoff scores, n (%)**					
	ASMMI^b^	5 (17)	4 (13)	.72	
	Dominant HGS^c^	21 (70)	28 (93)	.02^d^	
	Gait speed	28 (93)	29 (97)	.55	
**Frailty status, n (%)**					.19
	Robust	13 (43)	15 (50)		
	Prefrail	4 (13)	8 (27)		
	Frail	13 (43)	7 (23)		
**Sarcopenia status, n (%)**					.16^e^
	No	9 (30)	3 (10)		
	Possible	16 (53)	24 (80)		
	Confirmed	2 (7)	1 (3)		
	Severe	3 (10)	2 (7)		

^a^Mann-Whitney *U* test (continuous variables) or chi-square test (categorical variables).

^b^ASMMI: appendicular skeletal muscle mass index.

^c^HGS: handgrip strength.

^d^The *P* value met the threshold for significance (*P*<.05).

^e^Fisher exact test.

### Comparisons of Primary Outcomes Between the Intervention and Control Groups

A significant group×time interaction effect was observed in frailty status as measured by the Study of Osteoporotic Fractures index (*F*_2,116_=5.594; *P*=.007), with a small to medium effect size (η^2^=0.088) [45], indicating that the intervention group had a more significant reduction in frailty scores over time (Table 2). In terms of the sarcopenia diagnostic criteria, (1) body components showed notable improvements in appendicular skeletal muscle mass over time (*F*_2,58_=7.934; *P*=.005), with a significant group×time interaction (*F*_2,116_=9.895; *P*=.002), indicating larger gains in the intervention group. This trend was reflected in the ASMMI scores, revealing significant time (*F*_2,58_=7.339; *P*=.005) and group×time effects (*F*_2,116_=8.408; *P*=.003). Post hoc analysis indicated significant improvements from baseline to after the intervention and from midstudy to after the intervention in both appendicular skeletal muscle mass and ASMMI scores. (2) There was a significant increase in dominant HGS scores in the intervention group, with a notable interaction effect (*F*_2,116_=5.326; *P*=.01) and a small to medium effect size (η^2^=0.087). Post hoc analysis revealed significant improvements from baseline to after the intervention and from midstudy to after the intervention, exceeding the minimal clinically important difference (MCID). Lastly, (3) functional performance exhibited significant time effects in gait speed (ms; *F*_2,58_=6.664; *P*=.005), with a large effect size for the group×time interaction (η^2^=0.314), indicating that the intervention group experienced significantly greater improvements in walking speed. Post hoc analysis showed significant improvements from baseline to after the intervention, exceeding the MCID. The MCID signifies the smallest change in a clinical outcome measure perceived as meaningful by patients or clinicians [46]. The improvements in both HGS scores and walking speed exceeded the MCID, which is an indication of not only statistical significance but also clinical relevance after RFA training.

**Table 2:** Data of primary outcomes at baseline, midstudy (week 6), and after the intervention (week 12).

Primary outcomes				Baseline	Midstudy	After the intervention
**SOF^a^ index**						
	Intervention group, mean (SD)			1.07 (1.05)	1.00 (1.01)	0.80 (0.85)
	Control group, mean (SD)			0.73 (0.83)	0.80 (0.85)	0.80 (0.81)
	**2-way ANOVA**					
		**Time**				
			*F* test (*df*)	—^b^	—	2.650 (2,58)
			*P* value	—	—	.08
		**Group×time**				
			*F* test (*df*)	—	—	5.594 (2,116)
			*P* value	—	—	.007^c,d^
		Effect size (η^2^)		—	—	0.088
**Appendicular skeletal muscle mass (kg)**						
	Intervention group, mean (SD)			17.29 (3.81)	18.63 (4.31)	19.13 (4.58)
	Control group, mean (SD)			17.05 (2.95)	16.94 (2.81)	16.96 (2.40)
	**2-way ANOVA**					
		**Time**				
			*F* test (*df*)	—	—	7.934 (2,58)
			*P* value	—	—	.005^c^
		**Group×time**				
			*F* test (*df*)	—	—	9.895 (2,116)
			*P* value	—	—	.002^c,d^; .008^e^; .02^f^
		Effect size (η^2^)		—	—	0.165
**Appendicular skeletal muscle mass index (kg/m^2^)**						
	Intervention group, mean (SD)			7.37 (1.25)	7.92 (1.72)	8.15 (1.77)
	Control group, mean (SD)			7.17 (0.82)	7.12 (0.86)	7.15 (0.84)
	**2-way ANOVA**					
		**Time**				
			*F* test (*df*)	—	—	7.339 (2,58)
			*P* value	—	—	.005^c^
		**Group×time**				
			*F* test (*df*)	—	—	8.408 (2,116)
			*P* value	—	—	.003^c,d^; .009^e^; .03^f^
		Effect size (η^2^)		—	—	0.144
**Fat mass index (kg/m^2^)**						
	Intervention group, mean (SD)			7.41 (2.73)	6.98 (2.71)	6.62 (2.60)
	Control group, mean (SD)			9.00 (2.94)	8.71 (3.03)	8.38 (2.95)
	**2-way ANOVA**					
		**Time**				
			*F* test (*df*)	—	—	16.233 (2,58)
			*P* value	—	—	<.001^c^
		**Group×time**				
			*F* test (*df*)	—	—	0.241 (2,116)
			*P* value	—	—	.71
		Effect size (η^2^)		—	—	0.005
**Fat-free mass index (kg/m^2^)**						
	Intervention group, mean (SD)			16.96 (1.76)	17.10 (2.00)	17.67 (1.99)
	Control group, mean (SD)			16.85 (1.00)	16.86 (1.52)	17.26 (1.80)
	**2-way ANOVA**					
		**Time**				
			*F* test (*df*)	—	—	7.583 (2,58)
			*P* value	—	—	.002^c^
		**Group×time**				
			*F* test (*df*)	—	—	0.480 (2,116)
			*P* value	—	—	.57
		Effect size (η^2^)		—	—	0.010
**Dominant handgrip strength (kg; MCID^g^: 2.44-2.69 kg)**						
	Intervention group, mean (SD)^h^			14.51 (6.42)	17.60 (6.49)	20.80 (7.59)
	Control group, mean (SD)			13.40 (5.51)	15.16 (5.73)	16.20 (6.04)
	**2-way ANOVA**					
		**Time**				
			*F* test (*df*)	—	—	35.698 (2,58)
			*P* value	—	—	<.001^c^
		**Group×time**				
			*F* test (*df*)	—	—	5.326 (2,116)
			*P* value	—	—	.01^c,d^; <.001^e,i^; .001^f^
		Effect size (η^2^)		—	—	0.087
**Walking speed (ms; MCID: 0.1-0.2 ms)**						
	Intervention group, mean (SD)^h^			0.47 (0.25)	0.51 (0.31)	0.53 (0.33)
	Control group, mean (SD)			0.48 (0.23)	0.58 (0.30)	0.53 (0.27)
	**2-way ANOVA**					
		**Time**				
			*F* test (*df*)	—	—	6.664 (2,58)
			*P* value	—	—	.005^c^
		**Group×time**				
			*F* test (*df*)	—	—	25.173 (2,116)
			*P* value	—	—	<.001; .009^a^
		Effect size (η^2^)		—	—	0.314

^a^SOF: Study of Osteoporotic Fractures.

^b^Not applicable.

^c^*P* values that met the threshold for significance were analyzed using mixed repeated measures ANOVA with time as a within-participant factor and group as a between-participant factor.

^d^A post hoc Bonferroni test was performed to compare baseline, midstudy, and postintervention scores in the intervention group.

^e^Post hoc analysis showed significant differences between baseline and postintervention scores.

^f^Post hoc analysis showed significant differences between midstudy and postintervention scores.

^g^MCID: minimal clinically important difference.

^h^The improvements in the scores from baseline to after the intervention exceeded the MCID.

^i^Post hoc analysis showed significant differences between baseline and midstudy scores.

### Comparisons of Secondary Outcomes Between the Intervention and Control Groups

The secondary outcomes included muscle strength, morphology, hand dexterity, ADLs, HRQoL, and cognitive function. The intervention group had significant increases in the MVIC of the biceps and triceps brachii muscles, with time effects (biceps: *F_2,58_*=12.879; *P*<.001; triceps: *F_2,58_*=32.684; *P*<.001) and group×time interactions (biceps: *F_2,116_*=6.532; *P*=.004; triceps: *F_2,116_*=11.797; *P*<.001; Multimedia Appendix 3). Muscle thickness of the biceps brachii (measured using ultrasonography) also increased (*F_2,58_*=10.520; *P*<.001), with significant group×time interaction (*F_2,116_*=10.029; *P*<.001) in the intervention group. Hand dexterity improvements in the intervention group were significant over time (*F_2,58_*=16.848; *P*<.001), with a notable group×time interaction (*F_2,116_*=12.374; *P*<.001). ADLs, as measured by the Kihon checklist, showed no significant changes over time within the groups for physical function (*F_2,58_*=1.005; *P*=.34), but there were significant group×time interactions for both physical function (*F_2,116_*=5.599; *P*=.01) and mood function (*F_2,116_*=7.376; *P*=.003), indicating impactful intervention on ADLs. HRQoL, as assessed by SF-36 scores, demonstrated no significant changes over time in physical function (*F_2,58_*=0.667; *P*=.47) or in the interaction effect between group and time (*F_2,116_*=0.516; *P*=.54). However, improvements in mental function were observed (*F_2,58_*=6.571; *P*=.009) without significant differences between the groups.

In summary, the exergame-RFA intervention resulted in significant improvements in muscle strength (medium to large effect sizes), hand dexterity (large effect size), ADLs (medium effect size), and cognitive function (large effect) compared to the control group. However, the exergame-RFA intervention did not significantly enhance HRQoL or the thickness of the quadriceps and gastrocnemius muscles measured using ultrasonography compared to the control group. Summary of the significant group**×**time interactions of the outcome measures of this study are presented in Multimedia Appendix 4.

## Discussion

### Principal Findings

To our knowledge, this was the first study to evaluate the clinical effectiveness of RFA, an exergame that uniquely integrates aerobic and RT with real-time feedback in an engaging manner. Considering the multifaceted nature of frailty and sarcopenia and their varied definitions [12], our focus was on muscle and functional performance parameters. Our findings revealed that the exergame-RFA intervention significantly improved muscle mass, strength, and functional performance among older adult LTCF users compared to standard care. This underscores its potential as a novel therapeutic strategy to partially prevent and address frailty and sarcopenia in older adults.

This project stands out because it delivered an exergame-based exercise program using the RFA system. Similar to other exergames, RFA uses a gamified approach and immersive scenarios for player motivation through role-playing [47]. It offers visual feedback and sensory feedback from the screen and the Ring-Con, respectively. Unlike other exergames that mostly require fingertip operation, RFA, with its unique Ring-Con, demands significantly higher energy expenditure [48] and encompasses a multicomponent exercise regimen, blending PRT with aerobic activities for enhanced strength, balance, and muscle flexibility. Previous studies have confirmed the efficacy of PRT in improving physical function and strength among community-dwelling older adults due to its accessibility, low cost, and effectiveness [49,50]. Consistent with these findings, our results demonstrate that the exergame-RFA intervention can significantly enhance ASMMI, HGS, MVIC of the dominant upper extremity, and overall functional performance.

Another distinctive aspect of delivering exergames through RFA is its capability to determine the initial volume of exercise required at each stage, adjusting it progressively and automatically based on the player’s performance. At the start of the exergame-RFA intervention, participants were allowed to select their exercise intensity based on age, physical fitness, and preferred exercise intensity. This intensity setting in RFA is determined by a manufacturer-developed algorithm and follows a specific procedure: initially, users input their age and sex (step 1), followed by their exercise habits and desired exercise intensity (load; step 2). From these inputs, the load level (1-30) is automatically set on a scale from 1 (weakest) to 30 (strongest). As the load level increases, users need to apply more force on the Ring-Con to defeat enemies in the game, facilitating personalized exercise progression. This feature saves time and manpower by being machine adjustable [51]. However, for older adults (aged ≥65 y), the intensity options are limited, with the system defaulting to a maximum load setting of ≤15. While a preliminary study on older adults suggests that they can generally achieve moderate or greater intensity levels in RFA as assessed by the Karvonen heart rate reserve formula, it also recommended using established and validated exercise intensity indices (eg, heart rate and Borg Rating of Perceived Exertion Scale) alongside considering RFA’s intensity settings [51]. Furthermore, the ACSM recommends increasing intensity over time to maintain exercises at moderate levels (41%-60% of 1 repetition maximum for resistance exercise and Borg RPE 12-14 for aerobic exercise) [52]. Consequently, we set the target intensity of our exergame-based exercise at Borg RPE 13, leaving the weight intensity to the discretion of the Nintendo Switch. Although this approach is practical in real-world settings, and it is safer for older adults to perform PRT by squeezing or stretching the Ring-Con, a limitation remains in not being able to verify whether the Ring-Con’s weight intensity aligns with moderate levels.

Although they have the capacity to walk short distances for gait assessments, many older adults in LTCFs rely on wheelchairs for mobility (attributed to poor muscle endurance) [28]; hence, our RFA program incorporated a knee assist mode directly within the RFA system. This feature allowed us to focus on exercises for strengthening the upper extremities and trunk. This approach enabled participants to train in a seated position, thereby minimizing the risk of falls. Indeed, no participant in the intervention group experienced serious adverse effects. Consequently, it was logical that most outcomes of this study, including HGS, MVIC of the biceps and triceps brachii muscles, thickness of the biceps muscle measured using ultrasonography, and the box and block test, demonstrated significant group×time training effects.

In this study, we assessed both MVIC and muscle thickness measured using ultrasonography to determine whether early morphological changes in the trained muscles could be detected prematurely. This approach was taken because aging can dampen the hypertrophic response of muscle groups to RT [53]. Unlike in healthy young adults, where neural factors play a significant role in the initial increase in strength, and muscle hypertrophy becomes predominant after the first 3 to 5 weeks, the effects of muscle training in older adults may rely entirely on neuromuscular adaptation after an 8-week training course [54]. Interestingly, we observed significant improvements in biceps brachii muscle thickness measured using ultrasonography, rather than in MVIC, at the midstudy assessment. By the end of the intervention, both MVIC and biceps brachii muscle thickness measured using ultrasonography showed significant enhancements. However, no improvement in the thickness of lower leg muscles measured using ultrasonography was observed, despite a significant increase in walking speed after the intervention. This suggests that improvements in muscle parameters do not directly translate into functional performance enhancements. The relationship between muscle parameters and functional performance is complex and likely influenced by multiple factors.

Although the exergame-RFA intervention was focused on the training of upper extremities and the trunk, we found significant improvement in gait speed, one of the diagnostic criteria of sarcopenia. Walking encompasses complex movements requiring several functional tasks [55]. Participants had to maintain balance in various positions, such as leaning forward, reaching forward, and lateral shifting, to perform the exergame-RFA exercise prescription. Although our protocol did not permit training of the lower extremities, the movements in *arm fit skills* exercises likely engaged the trunk, hips, and knees. Granacher et al [56] demonstrated that the ability of older adults to rise from a chair, ambulate, and make turns improved after 9 weeks of core muscle strength training. Park et al [57] observed an increase in walking speed after a sitting boxing program for 6 weeks. Both studies showed that strengthening programs can induce adaptive processes, particularly in the neuromuscular system. These processes, in turn, enhance balance performance and functional mobility. Therefore, the observed increase in gait speed in our study was reasonable.

We observed significant improvements in ADLs, both in physical (Kihon checklist items 1-20) and mood (Kihon checklist items 21 to 25) aspects. Nevertheless, after the exergame-RFA intervention, we only noted a significant improvement in mental function, not in physical function, as measured by the SF-36. A systematic review involving 15 eligible randomized controlled trials indicated that exercise positively affects physical outcomes and enhances QoL and ADLs in older adults [58]. A large cohort study showed that community-dwelling older adults engaging in moderate to vigorous physical activity had a lower risk of ADL dependence [59]. Another study found a significant association between better physical function and higher levels of physical function as measured by the SF-36 [60]. Our results align with these findings to some extent. However, whether there is a strong correlation between muscle strength, muscle mass, functional performance, and these questionnaires still requires larger studies with longer follow-up periods to elucidate the association and even the causal relationship.

Exergames have been shown to have therapeutic effects on cognitive function among older adults [61,62]. Exergame environments enhance spatial awareness, challenging players with tasks that involve visual and auditory stimuli, cues, and feedback. Immersion into these virtual environments aids in attention restoration, stress reduction, and cognitive rehabilitation [62]. In addition, exergames necessitate decision-making during gameplay [61]. Therefore, it was not surprising to observe significant improvements in the brain health test score after the exergame-RFA intervention. Identifying a cutoff value for cognitive measurements to determine suitability for exergame participation among LTCF users would enhance the application of these findings.

### Limitations

Our study includes several limitations. First, while the sample size exceeded the minimum requirements for statistical analysis, it remained relatively small. Second, participants were recruited from nursing homes and day care centers in rural southern Taiwan, leading to a lower attrition rate than anticipated [63], potentially due to the unique characteristics of rural regions where older adults have limited health care options and may value services more highly. As a result, our findings may only be generalizable to similar populations. Third, the exergame-RFA intervention and evaluation period spanned only 3 months. Although improvements in muscle mass, strength, muscle thickness measured using ultrasonography, and certain questionnaire aspects were observed, this duration may have been too brief to detect significant increases in functional performance among the older adults. Fourth, there was no established protocol for training with RFA. Although the exercise prescription was based on ACSM guidelines for older adults, we cannot confirm the idealness of this exergame-based exercise protocol. The individualized setting and progression of exercises, a hallmark of using RFA for older adult training, were assessed solely through the Borg RPE scale, leaving uncertainties about the precise resistance offered by the Ring-Con during each session. Fourth, there was no established protocol for training with RFA. Although the exercise prescription was based on ACSM guidelines for older adults, we cannot confirm the idealness of this exergame-based exercise protocol. Furthermore, although the ability to automatically customize exercise settings and progression is a key feature of using RFA for training older adults, in this project, exercise intensity was only measured using the subjective Borg RPE scale. We were unable to verify the precise resistance provided by the Ring-Con during each training session. Finally, due to the small sample size, we did not perform a subgroup analysis to determine whether specific subgroups would benefit more from the exergame-RFA intervention. Future studies should aim to recruit larger samples to explore how older adults with varying degrees of frailty and sarcopenia respond to the exergame-RFA intervention. We also recommend further investigation into the optimal volume and long-term effects of the exergame-RFA intervention on older adults through larger randomized trials with control groups and extended follow-up periods, drawing from this study as a reference.

### Conclusions

In summary, our study contributes valuable insights into the practical application of exergame-based exercises within LTCF settings, affirming the potential of such interventions to improve muscle parameters; functional performance; and, by extension, HRQoL among older adults. These findings not only echo the sentiments expressed in the prestudy expectations but also pave the way for future research to refine and expand upon these interventions, ensuring that they meet the holistic needs of the aging population. Further studies could explore the integration of lower extremity exercises and more personalized exergame programs, aiming to encompass the full spectrum of sarcopenia criteria and ADL requirements.

## Appendix

Multimedia Appendix 1 CONSORT checklist.

Multimedia Appendix 2 Flowchart of the exergame-based multicomponent training program; exercise prescription of an exergame using Ring Fit Adventure based on the frequency, intensity, time, type, volume, and progression principle; and instruments and measures implemented for data collection in this study.

Multimedia Appendix 3 Data of secondary outcomes at baseline, midstudy (week 6), and after the intervention (week 12).

Multimedia Appendix 4 Summary of significant group×time effects on outcome measures.

## Abbreviations

ACSM: American College of Sports Medicine
ADL: activity of daily living
ASMMI: appendicular skeletal muscle mass index
CONSORT: Consolidated Standards of Reporting Trials
exergame-RFA: exergame-based exercise training program delivered via Ring Fit Adventure
EXPPLORE: Using an Exergame to Prevent and Postpone the Loss of Muscle Mass, Muscle Strength, and Functional Performance in Rural Elders
HGS: handgrip strength
HRQoL: health-related quality of life
LTCF: long-term care facility
MCID: minimal clinically important difference
MVIC: maximum voluntary isometric contraction
PRT: progressive resistance training
RFA: Ring Fit Adventure
RPE: rating of perceived exertion
RT: resistance training
SF-36: Short Form Health Survey-36
SPIRIT: Standard Protocol Items: Recommendations for Interventional Trials

## Data Availability

The data sets generated and analyzed during this study are available from the corresponding author on reasonable request.
