# Supplementary material for: Assessing the Clinical Effectiveness of an Exergame-Based Exercise Training Program Using Ring Fit Adventure to Prevent and Postpone Frailty and Sarcopenia Among Older Adults in Rural Long-Term Care Facilities: Randomized Controlled Trial
Source: J Med Internet Res. 2024 Jul 18;26:e59468. doi: 10.2196/59468 (PMC11294767; doi:10.2196/59468)
Supplement: Multimedia Appendix 2 [file jmir_v26i1e59468_app2.docx]

Multimedia Appendix 2. Flowchart of the exergame-based multicomponent training program; exercise prescription of an exergame using Ring Fit Adventure based on the frequency, intensity, time, type, volume, and progression principle; and instruments and measures implemented for data collection in this study.

Table S1. Flowchart of Exergame-based multicomponent training program

| Timeframe | Activity | Activity description | Mainly target muscles and joints |
| --- | --- | --- | --- |
| 0-10 minutes | Warm-up | Flexibility exercise. | Progressive static stretch of the neck, chest, arm, thighs, and legs. |
| 11-50 minutes | Main training | Ringfit Adventure with knee assist mode. The player controls the character in the Adventure Mode by squeezing and stretching the Ring-Con. | **Muscles**: trapezius, triceps, pectoralis major and minor, and core muscles.  **Joints**: shoulder horizontal adduction/ abduction, shoulder external/internal rotation, and elbow flexion |
|  |  | To defeat the monsters in each stage, the players has to use the combination of the following six arm fit skills. | Not applicable |
| 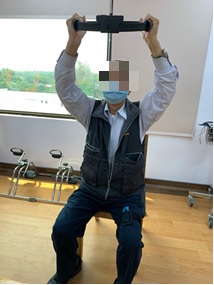 | | **Overhead press**: Hold the Ring-Con overhead and squeeze it. | **Muscles**: deltoid, and biceps brachii  **Joints**: shoulder flexion, and elbow flexion/extension |
| 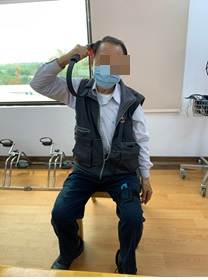 | | **Shoulder press**: Hold the Ring-Con on each side of the shoulder and squeeze it. | **Muscles**: biceps/triceps brachii  **Joints**: elbow flexion/extension |
| 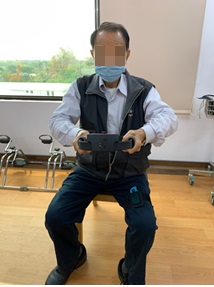 | | **Front press**: Hold the Ring-Con below umbilicus with anterior-tilting of the trunk slightly. | **Muscles**: trapezius, triceps, pectoralis major and minor, and core muscles.  **Joints**: shoulder horizontal adduction/ abduction, and elbow flexion |
| 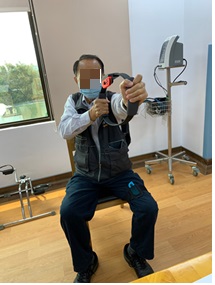 | | **Bow pull**: Put the Ring-Con in front of the chest and pull it like it’s a bow. | **Muscles**: biceps/triceps brachii, and latissimus dorsi  **Joints**: shoulder adduction/ abduction, elbow flexion/flexion, and wrist flexion/extension |
| 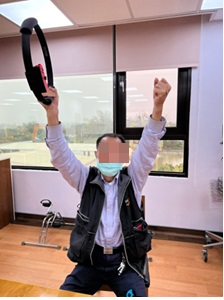 | | **Overhead arm spin**: raise both arms straight up and twist the wrists. | **Muscles**: deltoid and shoulder girdles  **Joints**: shoulder flexion, elbow pronation/supination, and wrist flexion |
| 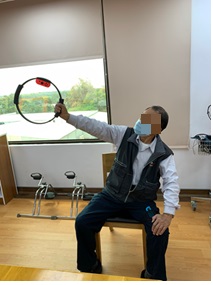 | | **Tricep kickback**: With elbow locked, move the Ring-Con up-and-down | **Muscles**: triceps, and spinal erectors  **Joints**: shoulder horizontal adduction/abduction, and elbow flexion/extension |
| 51-60 minutes | Cool-down | Flexibility exercise. | Progressive static stretch of the neck, chest, arm, thighs, and legs. |

Table S2. Exercise prescription of exergame via Ring Fit Adventure using the FITT-VP principle

| **Frequency** | The exergame-RFA regimen was scheduled bi-weekly, with each session spaced at least 48 hours apart. |
| --- | --- |
| **Intensity** | The target intensity of the exergame-RFA regimen was set at a level of 13 (somewhat hard) on the Borg Rating of Perceived Exertion scale. |
| **Time** | Each exergame-RFA session spanned 50 minutes, consisting of 10 minutes dedicated to warm-up and cool-down respectively, with a main 30-minute exercise segment. |
| **Type** | During the game, players' movements were detected through a Pilates ring (Ring-Con) and a strap equipped with a controller (Joy-Con). In Adventure Mode, players earned coins by stretching and squeezing the Ring-Con. In Battle Mode, players must utilize various fit skills to defeat monsters. Thus, exergame-RFA regimen could be considered a multi-component exercise regimen that combines resistance and aerobic exercises, incorporating elements of strength training, balance, and muscle stretching. |
| **Duration** | The exergame-RFA regimen lasted for a total of 12 weeks. |
| **Progression** | Upon the first time of the game, individual data such as age, gender, and weight were entered. Players were then encouraged to apply maximum force to the Ring-Con, akin to determining a one-repetition maximum in traditional resistance training. Based on this initial input, the game set and subsequently auto-adjusted the exercise quantities per stage, tailored to each player's performance.  After each training session, the Joy-Con attached to the Ring-Con detected the player's heart rate to assess whether the target intensity of the exercise prescription was reached, automatically increasing the intensity if necessary. The higher the intensity, the more repetitions required and the greater resistance of the Ring-Con to stretch and squeeze. |

Table S3. Instruments and measures implemented for data collection of this study

| **Instrument or Measure** | **Outcome** | **Description** | **Time point** | **Reference** |
| --- | --- | --- | --- | --- |
| Appendicular skeletal muscle mass index | Diagnostic criterion of sarcopenia. Primary outcome | Measured by bioelectrical impedance analysis. Defined as the appendicular skeletal muscle mass (Kg) divided by the height squared (m^2^) | T0, T1, T2 | [29] |
| Dominant handgrip strength | Diagnostic criterion of sarcopenia. Primary outcome | Measured by JAMAR dynamometer under standard position. | T0, T1, T2 | [30] |
| Gait speed | Diagnostic criterion of sarcopenia. Primary outcome | The participants are asked to walk at a normal speed on a 6-m long corridor without a barrier and the usual gait speed calculated by measuring the time spent by a participant. | T0, T1, T2 | [29] |
| Study of osteoporotic fracture index | Indicator of frailty. Primary outcome | Including three components: (a) a weight loss of ≥5% during the preceding year, (b) an inability to rise from a chair five times without using the arms, and (c) an answer of ‘no’ to the question ‘Do you feel full of energy?’ | T0, T1, T2 | [31] |
| Box and block Test | Indicator of hand dexterity. Secondary outcome | The number of blocks the participants transferred in 60 seconds from one compartment to the other compartment of the wooden box. | T0, T1, T2 | [32] |
| Biceps and triceps brachii muscle strength of the dominant side | Indicator of muscle strength. Secondary outcome | Using the microFET® 3 to measure the maximal voluntary isometric contraction under standard positions. | T0, T1, T2 | [33] |
| Sonographic thickness of biceps, quadriceps, and gastrocnemius muscles | Indicator of muscle morphjology. Secondary outcome | Using a portable LOGIQ e ultrasound, equipped with a 5-12 MHz linear array transducer, to measure the muscle thickness under standard positions. | T0, T1, T2 | [34-36] |
| Range of motion of the joints of upper extremity | Indicator of functional movement. Secondary outcome | Using a goniometer under standard positions to measure shoulder flexion, abduction, and external rotation; elbow flexion and extension; forearm supination and pronation and wrist flexion and extension. | T0, T1, T2 | [37] |
| Kihon checklist-Taiwan | Indicator of general function. Secondary outcome | A self-reported questionnaire, consisting of 25 items divided into 7 sub-categories. Each item is rated as pass (0) or fail (1). A higher total score indicates a lower level of function. | T0, T1, T2 | [38] |
| Medical outcomes study 36-Item short-form health survey | Indicator of health-related quality of life. Secondary outcome | A self-assessment containing 36 items, divided into 8 subscales. Responses to each question were transformed to a scale ranging from 0–100. The higher the scores, the better the quality of life. | T0, T1, T2 | [39] |
| Brain health test brief cognitive test | Indicator of cognitive function. Secondary outcome | A clinical assessment tool, including orientation to time, immediate and delayed recall of five items, categorical verbal fluency test (listing four-legged animals in one minute), and the Clock Drawing Test. The higher the scores, the better the cognitive function. | T0, T1, T2 | [40] |

T0, baseline measurement; T1, the end of the sixth week after the exergame-based exercise; T2, the end of the 12^th^ week after the exergame-based exercise
