# Supplementary material for: Assessing the Clinical Effectiveness of an Exergame-Based Exercise Training Program Using Ring Fit Adventure to Prevent and Postpone Frailty and Sarcopenia Among Older Adults in Rural Long-Term Care Facilities: Randomized Controlled Trial
Source: J Med Internet Res. 2024 Jul 18;26:e59468. doi: 10.2196/59468 (PMC11294767; doi:10.2196/59468)
Supplement: Multimedia Appendix 3 [file jmir_v26i1e59468_app3.ocx]

Multimedia Appendix 3. Data of secondary outcomes at baseline, midstudy (week 6), and after the intervention (week 12).

| Secondary outcomes | | | | | Baseline | Midstudy | After the intervention |
| --- | --- | --- | --- | --- | --- | --- | --- |
| **Maximum voluntary isometric contraction** | | | | | | | |
|  | **Biceps brachii muscle (kg)** | | | | | | |
|  |  | Intervention group, mean (SD) | | | 2.89 (0.52) | 3.14 (0.64) | 3.62 (0.73) |
|  |  | Control group, mean (SD) | | | 2.92 (0.83) | 2.95 (0.83) | 3.04 (0.82) |
|  |  | **2-way ANOVA** | | | | | |
|  |  |  | **Time** | | | | |
|  |  |  |  | *F* test (*df*) | —^a^ | — | 12.879 (2,58) |
|  |  |  |  | *P* value | — | — | <.001^b^ |
|  |  |  | **Group×time** | | | | |
|  |  |  |  | *F* test (*df*) | — | — | 6.532 (2,116) |
|  |  |  |  | *P* value | — | — | .004^b,c^; <.001^d^; <.001^e^ |
|  |  |  | Eﬀect size (ɳ^2^) | | — | — | 0.108 |
|  | **Triceps brachii muscle (kg)** | | | | | | |
|  |  | Intervention group, mean (SD) | | | 3.00 (0.68) | 3.24 (0.61) | 3.96 (0.63) |
|  |  | Control group, mean (SD) | | | 3.03 (0.69) | 2.93 (0.56) | 3.23 (0.87) |
|  |  | **2-way ANOVA** | | | | | |
|  |  |  | **Time** | | | | |
|  |  |  |  | *F* test (*df*) | — | — | 32.684 (2,56) |
|  |  |  |  | *P* value | — | — | <.001^b^ |
|  |  |  | **Group×time** | | | | |
|  |  |  |  | *F* test (*df*) | — | — | 11.797 (2,116) |
|  |  |  |  | *P* value | — | — | <.001^b,c^; <.001^d^; <.001^e^ |
|  |  |  | Eﬀect size (ɳ^2^) | | — | — | 0.179 |
| **Muscle thickness measured using ultrasonography** | | | | | | | |
|  | **Biceps brachii muscle (cm)** | | | | | | |
|  |  | Intervention group, mean (SD) | | | 2.02 (0.59) | 2.13 (0.55) | 2.41 (0.70) |
|  |  | Control group, mean (SD) | | | 2.27 (0.44) | 2.34 (0.37) | 2.29 (0.36) |
|  |  | **2-way ANOVA** | | | | | |
|  |  |  | **Time** | | | | |
|  |  |  |  | *F* test (*df*) | — | — | 10.520 (2,56) |
|  |  |  |  | *P* value | — | — | <.001 |
|  |  |  | **Group×time** | | | | |
|  |  |  |  | *F* test (*df*) | — | — | 10.029 (2,116) |
|  |  |  |  | *P* value | — | — | <.001^b,c^; .001^d^; .04^e^; .02^f^ |
|  |  |  | Eﬀect size (ɳ^2^) | | — | — | 0.167 |
|  | **Quadriceps muscle (cm)** | | | | | | |
|  |  | Intervention group, mean (SD) | | | 2.36 (0.67) | 2.51 (0.68) | 2.64 (0.71) |
|  |  | Control group, mean (SD) | | | 2.32 (0.48) | 2.38 (0.57) | 2.47 (0.55) |
|  |  | **2-way ANOVA** | | | | | |
|  |  |  | **Time** | | | | |
|  |  |  |  | *F* test (*df*) | — | — | 16.710 (2,58) |
|  |  |  |  | *P* value | — | — | <.001 |
|  |  |  | **Group×time** | | | | |
|  |  |  |  | *F* test (*df*) | — | — | 1.760 (2,116) |
|  |  |  |  | *P* value | — | — | .19 |
|  |  |  | Eﬀect size (ɳ^2^) | | — | — | 0.034 |
|  | **Gastrocnemius muscle (cm)** | | | | | | |
|  |  | Intervention group, mean (SD) | | | 1.12 (0.46) | 1.26 (0.45) | 1.34 (0.49) |
|  |  | Control group, mean (SD) | | | 1.06 (0.34) | 1.14 (0.39) | 1.11 (0.40) |
|  |  | **2-way ANOVA** | | | | | |
|  |  |  | **Time** | | | | |
|  |  |  |  | *F* test (*df*) | — | — | 9.580 (2,58) |
|  |  |  |  | *P* value | — | — | .001^b^ |
|  |  |  | **Group×time** | | | | |
|  |  |  |  | *F* test (*df*) | — | — | 3.211 (2,116) |
|  |  |  |  | *P* value | — | — | .06 |
|  |  |  | Eﬀect size (ɳ^2^) | | — | — | 0.060 |
| **Hand dexterity** | | | | | | | |
|  | **Box and block test (MCID^g^: 5.5 cubes/min)** | | | | | | |
|  |  | Intervention group, mean (SD)^h^ | | | 49.82 (13.21) | 54.55 (13.86) | 56.96 (12.77) |
|  |  | Control group, mean (SD) | | | 43.21 (14.98) | 44.29 (14.71) | 43.60 (14.52) |
|  |  | **2-way ANOVA** | | | | | |
|  |  |  | **Time** | | | | |
|  |  |  |  | *F* test (*df*) | — | — | 16.848 (2,58) |
|  |  |  |  | *P* value | — | — | <.001^b^ |
|  |  |  | **Group×time** | | | | |
|  |  |  |  | *F* test (*df*) | — | — | 12.374 (2,116) |
|  |  |  |  | *P* value | — | — | <.001^b,c^; <.001^d^; <.001^f^ |
|  |  |  | Eﬀect size (ɳ^2^) | | — | — | 0.186 |
| **Activities of daily living** | | | | | | | |
|  | **Kihon checklist score (items 1-20)** | | | | | | |
|  |  | Intervention group, mean (SD) | | | 8.80 (4.29) | 8.70 (4.19) | 8.46 (4.03) |
|  |  | Control group, mean (SD) | | | 10.57 (3.82) | 10.60 (3.88) | 10.70 (3.82) |
|  |  | **2-way ANOVA** | | | | | |
|  |  |  | **Time** | | | | |
|  |  |  |  | *F* test (*df*) | — | — | 1.005 (2,58) |
|  |  |  |  | *P* value | — | — | .34 |
|  |  |  | **Group×time** | | | | |
|  |  |  |  | *F* test (*df*) | — | — | 5.599 (2,116) |
|  |  |  |  | *P* value | — | — | .01^b^ |
|  |  |  | Eﬀect size (ɳ^2^) | | — | — | 0.088 |
|  | **Kihon checklist score (items 21-25)** | | | | | | |
|  |  | Intervention group, mean (SD) | | | 1.50 (1.00) | 1.33 (1.09) | 1.20 (1.06) |
|  |  | Control group, mean (SD) | | | 1.43 (1.19) | 1.60 (1.22) | 1.63 (1.22) |
|  |  | **2-way ANOVA** | | | | | |
|  |  |  | **Time** | | | | |
|  |  |  |  | *F* test (*df*) | — | — | 0.379 (2,58) |
|  |  |  |  | *P* value | — | — | .63 |
|  |  |  | **Group×time** | | | | |
|  |  |  |  | *F* test (*df*) | — | — | 7.376 (2,116) |
|  |  |  |  | *P* value | — | — | .003^b^ |
|  |  |  | Eﬀect size (ɳ^2^) | | — | — | 0.113 |
|  | **Kihon checklist score (total)** | | | | | | |
|  |  | Intervention group, mean (SD) | | | 10.30 (4.89) | 10.03 (4.70) | 9.67 (4.43) |
|  |  | Control group, mean (SD) | | | 12.00 (4.70) | 12.20 (4.76) | 12.33 (4.64) |
|  |  | **2-way ANOVA** | | | | | |
|  |  |  | **Time** | | | | |
|  |  |  |  | *F* test (*df*) | — | — | 0.859 (2,58) |
|  |  |  |  | *P* value | — | — | .38 |
|  |  |  | **Group×time** | | | | |
|  |  |  |  | *F* test (*df*) | — | — | 8.093 (2,116) |
|  |  |  |  | *P* value | — | — | .003^b^ |
|  |  |  | Eﬀect size (ɳ^2^) | | — | — | 0.122 |
| **Health-related quality of life** | | | | | | | |
|  | **SF-36^i^ physical function** | | | | | | |
|  |  | Intervention group, mean (SD) | | | 65.23 (30.83) | 67.20 (30.36) | 67.80 (30.58) |
|  |  | Control group, mean (SD) | | | 60.86 (28.00) | 40.66 (29.66) | 61.17 (29.41) |
|  |  | **2-way ANOVA** | | | | | |
|  |  |  | **Time** | | | | |
|  |  |  |  | *F* test (*df*) | — | — | 0.667 (2,58) |
|  |  |  |  | *P* value | — | — | .47 |
|  |  |  | **Group×time** | | | | |
|  |  |  |  | *F* test (*df*) | — | — | 0.516 (2,116) |
|  |  |  |  | *P* value | — | — | .54 |
|  |  |  | Eﬀect size (ɳ^2^) | | — | — | 0.009 |
|  | **SF-36 mental function** | | | | | | |
|  |  | Intervention group, mean (SD) | | | 64.47 (25.87) | 70.60 (24.72) | 72.50 (25.51) |
|  |  | Control group, mean (SD) | | | 69.79 (25.51) | 73.34 (22.52) | 74.21 (22.68) |
|  |  | **2-way ANOVA** | | | | | |
|  |  |  | **Time** | | | | |
|  |  |  |  | *F* test (*df*) | — | — | 6.571 (2,58) |
|  |  |  |  | *P* value | — | — | .009^b^ |
|  |  |  | **Group×time** | | | | |
|  |  |  |  | *F* test (*df*) | — | — | 0.534 (2,116) |
|  |  |  |  | *P* value | — | — | .50 |
|  |  |  | Eﬀect size (ɳ^2^) | | — | — | 0.009 |
|  | **SF-36 total** | | | | | | |
|  |  | Intervention group, mean (SD) | | | 64.87 (26.35) | 68.90 (26.30) | 70.15 (26.85) |
|  |  | Control group, mean (SD) | | | 65.33 (26.10) | 67.00 (25.23) | 67.69 (25.15) |
|  |  | **2-way ANOVA** | | | | | |
|  |  |  | **Time** | | | | |
|  |  |  |  | *F* test (*df*) | — | — | 4.481 (2,58) |
|  |  |  |  | *P* value | — | — | .03^b^ |
|  |  |  | **Group×time** | | | | |
|  |  |  |  | *F* test (*df*) | — | — | 0.682 (2,116) |
|  |  |  |  | *P* value | — | — | .43 |
|  |  |  | Eﬀect size (ɳ^2^) | | — | — | 0.012 |
| **Cognitive function** | | | | | | | |
|  | **Brain health test score** | | | | | | |
|  |  | Intervention group, mean (SD) | | | 9.17 (4.22) | 10.17 (4.14) | 10.90 (3.96) |
|  |  | Control group, mean (SD) | | | 10.02 (4.56) | 9.60 (4.43) | 9.33 (4.65) |
|  |  | **2-way ANOVA** | | | | | |
|  |  |  | **Time** | | | | |
|  |  |  |  | *F* test (*df*) | — | — | 2.824 (2,58) |
|  |  |  |  | *P* value | — | — | .09 |
|  |  |  | **Group×time** | | | | |
|  |  |  |  | *F* test (*df*) | — | — | 15.285 (2,116) |
|  |  |  |  | *P* value | — | — | <.001^b^ |
|  |  |  | Eﬀect size (ɳ^2^) | | — | — | 0.211 |
