# Supplementary material for: Assessing the Clinical Effectiveness of an Exergame-Based Exercise Training Program Using Ring Fit Adventure to Prevent and Postpone Frailty and Sarcopenia Among Older Adults in Rural Long-Term Care Facilities: Randomized Controlled Trial
Source: J Med Internet Res. 2024 Jul 18;26:e59468. doi: 10.2196/59468 (PMC11294767; doi:10.2196/59468)
Supplement: Multimedia Appendix 4 [file jmir_v26i1e59468_app4.docx]

**Multimedia Appendix 4. Summary of significant group×time effects on outcome measures.**

| **Outcome** | **Indicator** | **Item** | **Significant Effect**  **Time x Group** |
| --- | --- | --- | --- |
| **Primary** | Frailty | Study of Osteoporotic Fractures Index | V |
|  | Sarcopenia | Appendicular skeletal muscle mass index | V |
|  |  | Handgrip strength | V |
|  |  | Gait speed | V |
| **Secondary** | Muscle strength | Biceps | V |
|  |  | Triceps | V |
|  | Sonographic muscle morphology | Biceps | V |
|  |  | Quadriceps |  |
|  |  | Gastrocnemius |  |
|  | Dexterity | Box and block test | V |
|  | Activity of Daily Living | Kihon checklist | V |
|  | Health-related Quality of Life | SF-36 |  |
|  | Cognition | Brain health test | V |
